# Development of a ten-signature classifier using a support vector machine integrated approach to subdivide the M1 stage into M1a and M1b stages of nasopharyngeal carcinoma with synchronous metastases to better predict patients' survival

**DOI:** 10.18632/oncotarget.6436

**Published:** 2015-11-30

**Authors:** Rou Jiang, Rui You, Xiao-Qing Pei, Xiong Zou, Meng-Xia Zhang, Tong-Min Wang, Rui Sun, Dong-Hua Luo, Pei-Yu Huang, Qiu-Yan Chen, Yi-Jun Hua, Lin-Quan Tang, Ling Guo, Hao-Yuan Mo, Chao-Nan Qian, Hai-Qiang Mai, Ming-Huang Hong, Hong-Min Cai, Ming-Yuan Chen

**Affiliations:** ^1^ Department of Nasopharyngeal Carcinoma, Sun Yat-sen University Cancer Center, Guangzhou, P. R. China; ^2^ Collaborative Innovation Center for Cancer Medicine, State Key Laboratory of Oncology in South China, Sun Yat-sen University Cancer Center, Guangzhou, P. R. China; ^3^ Department of Cancer Prevention, Sun Yat-sen University Cancer Center, Guangzhou, P. R. China; ^4^ Department of Ultrasonography, Sun Yat-sen University Cancer Center, Guangzhou, P. R. China; ^5^ Department of Clinical Trials Center, Sun Yat-sen University Cancer Center, Guangzhou, P. R. China; ^6^ School of Computer and Engineering, South China University of Technology, Guangzhou, P. R. China

**Keywords:** nasopharyngeal carcinoma, synchronous metastases, support vector machine, prognosis, therapy

## Abstract

The aim of this study was to develop a prognostic classifier and subdivided the M1 stage for nasopharyngeal carcinoma patients with synchronous metastases (mNPC). A retrospective cohort of 347 mNPC patients was recruited between January 2000 and December 2010. Thirty hematological markers and 11 clinical characteristics were collected, and the association of these factors with overall survival (OS) was evaluated. Advanced machine learning schemes of a support vector machine (SVM) were used to select a subset of highly informative factors and to construct a prognostic model (mNPC-SVM). The mNPC-SVM classifier identified ten informative variables, including three clinical indexes and seven hematological markers. The median survival time for low-risk patients (M1a) as identified by the mNPC-SVM classifier was 38.0 months, and survival time was dramatically reduced to 13.8 months for high-risk patients (M1b) (*P* < 0.001). Multivariate adjustment using prognostic factors revealed that the mNPC-SVM classifier remained a powerful predictor of OS (M1a vs. M1b, hazard ratio, 3.45; 95% CI, 2.59 to 4.60, *P* < 0.001). Moreover, combination treatment of systemic chemotherapy and loco-regional radiotherapy was associated with significantly better survival outcomes than chemotherapy alone (the 5-year OS, 47.0% vs. 10.0%, *P* < 0.001) in the M1a subgroup but not in the M1b subgroup (12.0% vs. 3.0%, *P* = 0.101). These findings were validated by a separate cohort. In conclusion, the newly developed mNPC-SVM classifier led to more precise risk definitions that offer a promising subdivision of the M1 stage and individualized selection for future therapeutic regimens in mNPC patients.

## INTRODUCTION

Nasopharyngeal carcinoma (NPC) is a distinct malignancy of the head and neck with a high incidence (4%-6%) of distant metastases [1-3]. Metastatic NPC is incurable and devastating, with a median survival of ten to fifteen months using palliative systemic chemotherapy[4]. The current American Joint Committee on Cancer (AJCC/UICC) Tumor, Node, and Metastasis (TNM) staging uses “M1” to denote the TNM stage for all patients with distant metastasis. Therefore, TNM staging ignores the heterogeneity between patients, and this system may exhibit reduced critical accuracy in patients with distant metastases [1, 5, 6]. NPC patients exhibit common clinical characteristics, such as tumor volume and number, and numerous molecular and hematological markers are also useful to predict outcomes of NPC patients, especially the microcosmic aspect of patients. Compared with molecular markers, hematological markers are more easily applicable to clinical practice because of its convenience and availability.

Palliative chemotherapy benefits the survival of newly diagnosed NPC patients with synchronous distant metastases (mNPC). Previous studies reported improved overall survival (OS) with loco-regional radiotherapy combined with chemotherapy in mNPC patients compared with chemotherapy alone [7]. However, the TNM classification system does not identify which patients should undergo palliative chemotherapy alone or combination treatment. Consequently, it is difficult for clinicians to choose the most suitable treatment for mNPC patients.

The above-mentioned facts prompted us to develop a novel prognostic model based on the clinical characteristics and hematological markers for mNPC patients to correctly predict the survival of mNPC patients and aid clinicians in treatment planning.

## RESULTS

### Patient characteristics

The baseline of patients' clinical features of the primary cohort and validation cohort were displayed in Table 1. In the primary cohort, patients ranged from 13 to 78 years old (median age, 48 years), and 80% were male. Bone was the most common site of metastasis (66.0%), followed by liver (38.0%) and lung (20.0%). Most patients exhibited multiple sites of metastases at the time of diagnosis (81.0%). The median survival was 22.6 months (range, 3.2–164.3 months). A total of 269 (78.0%) of the 347 patients had died by the last follow-up. The OS rates at 1, 2, 3 and 5 years were 76%, 46%, 31%, and 22%, respectively (data not shown).

**Table 1:** Clinical characteristics

Parameters	Primary cohort	Validation cohort
	n=347	n=106
Sex		
Female	69	23
Male	278	83
Age		
< 48 years	173	55
≥ 48 years	174	51
T Classification*		
T1-2	142	22
T3-4	205	84
N Classification*		
N0-1	134	25
N2-3	213	81
No. of metastatic organs		
Single	242	81
Multiple	105	25
No. of metastatic lesions		
Single	65	34
Multiple	282	72
Oligometastases		
No	132	44
Yes	215	62
Bony metastasis		
Absent	118	32
Present	229	74
Liver metastasis		
Absent	216	73
Present	131	33
Lung metastasis		
Absent	278	82
Present	69	24
Extraregional lymph node metastasis		
Absent	299	100
Present	48	6
Anticancer treatment		
Systemic chemotherapy alone	161	40
Chemotherapy plus radiotherapy	174	65
Radiotherapy alone	12	1

* According to Union for International Cancer Control /American Joint Committee on Cancer (UICC/AJCC) TNM classification system (6th edition, 2002).

### Combinational prognostic model via RFE-SVM analysis

**Figure 1:**
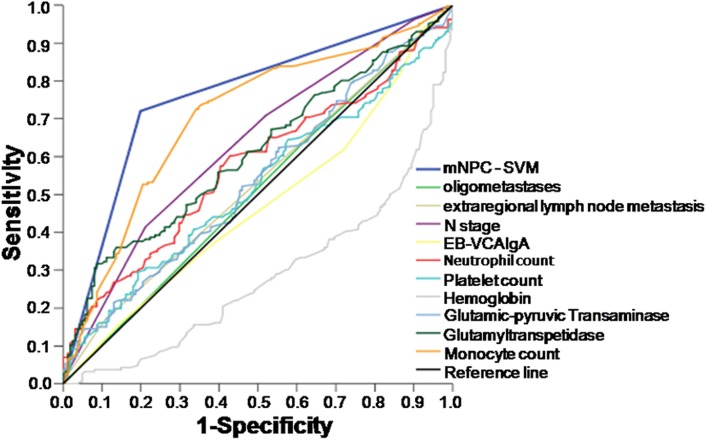
Receiver operating characteristic (ROC) curves for each of the selected ten variables and their combination, the mNPC-SVM classifier, as predictors of death as a result of mNPC within 2 years

Experiments of the entire dataset using RFE-SVM analysis identified ten informative variables, including three clinical indexes (oligometastases, N stage, and extraregional lymph node metastasis) and seven hematological markers (EB-VCA IgA, neutrophil count, monocyte count, platelet count, hemoglobin, glutamic-pyruvic transaminase, and glutamyltranspetidase). The ROC curves for each of the ten selected variables and their combination, the mNPC-SVM classifier, illustrated the maximum area under the curve (AUC) for each factor. The AUC for the mNPC-SVM classifier was significantly greater than the AUCs for all other individual prognostic factors (0.761, Figure 1). The specificity and sensitivity of mNPC-SVM were 71.3% and 80.7%, respectively. Significant association was observed between the mNPC-SVM and N stage, metastatic organ/lesion involvement and oligometastases by Spearman correlation analysis (Table 2). In the validation cohort, the AUC for the mNPC-SVM classifier was 0.633 (Supplemental Figure 1).

**Table 2:** Association of mNPC -SVM classifier with clinicopathologic characteristics

Parameters	mNPC -SVM classifier		r value	*p* value
	Predicted low risk	Predicted high risk		
Sex				
Female	38	31	−0.026	0.627
Male	144	134		
Age				
< 48 years	95	78	0.049	0.361
≥ 48 years	87	87		
T Classification*				
T1-2	76	66	0.018	0.740
T3-4	106	99		
N Classification*				
N0-1	88	46	0.21	<0.001
N2-3	94	119		
No. of metastatic organs				
Single	142	100	0.189	<0.001
Multiple	40	65		
No. of metastatic lesions				
Single	49	16	0.220	<0.001
Multiple	133	149		
Oligometastases				
No	56	76	−0.157	0.003
Yes	126	89		
Bony metastasis				
Absent	67	51	0.062	0.248
Present	115	114		
Liver metastasis				
Absent	122	94	0.104	0.054
Present	60	71		
Lung metastasis				
Absent	147	131	0.017	0.749
Present	35	34		
Extraregional lymph node metastasis				
Absent	161	138	0.070	0.195
Present	21	27		

* According to Union for International Cancer Control /American Joint Committee on Cancer (UICC/AJCC) TNM classification system (6th edition, 2002).

### Subdivision of the M1 stage using the mNPC-SVM classifier and overall survival

**Figure 2:**
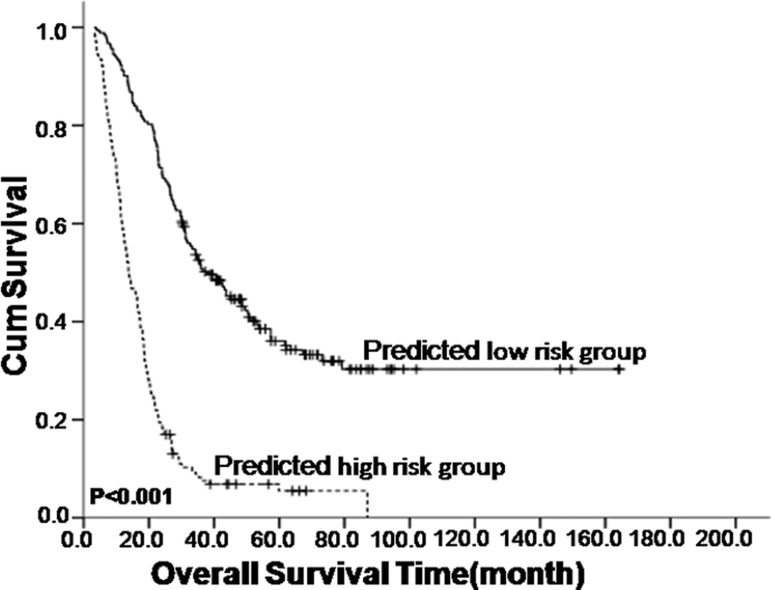
Kaplan–Meier survival analysis of the mNPC-SVM classifier in nasopharyngeal carcinoma patients with synchronous metastases (mNPC)

The mNPC-SVM classifier identified 182 patients as low risk (called the M1a stage) and 165 patients as high risk (defined as the M1b stage). The 2-year overall survival rates differed significantly between M1a and M1b patients (71.4% v 18.8%, *P* < 0.001). The corresponding overall survival also differed significantly. The median survival time for the low-risk patient group was 38.0 months, and survival time for the high-risk patient group was dramatically reduced to 13.8 months (*P* < 0.001, Figure 2). A similar trend was also observed in the validation cohort (*P* = 0.001, Supplemental Figure 2).

### Selection of independent prognostic factors of survival

Univariate analysis of the 347 mNPC patients using the mNPC-SVM classifier revealed an apparent association of 5 clinical indexes with OS (Table 3). The mNPC-SVM classifier remained a powerful predictor of OS after multivariate adjustment using clinicopathological characteristics (predicted low risk vs. predicted high risk, hazard ratio, 3.45; 95% CI, 2.59 to 4.60; *P* < 0.001). By contrast, there was no significant difference in OS after multivariate adjustment by N stage, metastatic organ/lesion involvement, liver metastasis or extraregional lymph node metastasis (Table 4).

**Table 3:** Univariate analysis of clinicopathological and hematological characteristics

Variables	Patients (N =347)	2-Year OS (95% CI)	Unadjusted HR (95%CI)	P
mNPC -SVM classifier				
Predicted low risk	182.00	0.71 (0.51-0.91)	Reference	
Predicted high risk	165.00	0.19 (0.17, 0.21)	3.71 (2.88, 4.79)	<0.001
Sex				
Male	278.00	0.46 (0.40, 0.52)	Reference	
Female	69.00	0.48 (0.34, 0.62)	0.84 (0.62, 1.14)	0.269
Age				
< 48 years	173.00	0.48 (0.39, 0.57)	Reference	
≥ 48 years	174.00	0.45 (0.37, 0.53)	1.07 (0.84, 1.36)	0.592
T Classification*				
T1-2	142.00	0.46 (0.37, 0.55)	Reference	
T3-4	205.00	0.46 (0.39, 0.53)	0.94 (0.74, 1.20)	0.626
N Classification*				
N0-1	134.00	0.57 (0.44, 0.70)	Reference	
N2-3	213.00	0.39 (0.33, 0.45)	1.38 (1.21, 1.57)	<0.001
No. of metastatic organs				
Single	242.00	0.53 (0.44, 0.62)	Reference	
Multiple	105.00	0.30 (0.25, 0.35)	1.72 (1.33, 2.21)	<0.001
No. of metastatic lesions				
Single	65.00	0.60 (0.38, 0.82)	Reference	
Multiple	282.00	0.43 (0.37, 0.49)	1.82 (1.31, 2.55)	<0.001
Oligometastases				
No	132.00	0.48 (0.38, 0.58)	Reference	
Yes	215.00	0.46 (0.39, 0.53)	0.92 (0.72, 1.18)	0.530
Bony metastasis				
Absent	118.00	0.45 (0.35, 0.55)	Reference	
Present	229.00	0.47 (0.40, 0.54)	0.89 (0.69, 1.14)	0.359
Liver metastasis				
Absent	216.00	0.53 (0.44, 0.62)	Reference	
Present	131.00	0.35 (0.39, 0.41)	1.56 (1.22, 1.99)	<0.001
Lung metastasis				
Absent	278.00	0.46 (0.40, 0.52)	Reference	
Present	69.00	0.46 (0.31, 0.61)	1.11 (0.82, 1.47)	0.530
Extraregional lymph node metastasis				
Absent	299.00	0.48 (0.41, 0.55)	Reference	
Present	48.00	0.36 (0.25, 0.47)	1.21 (1.03, 1.43)	0.022
EB-VCAIgA				
≤1:160	116.00	0.39 (0.32, 0.46)	Reference	
>1:160	231.00	0.50 (0.42, 0.58)	0.97 (0.75, 1.25)	0.790
EB-EAIgA				
≤1:10	106.00	0.42 (0.33, 0.51)	Reference	
>1:10	241.00	0.49 (0.42, 0.56)	0.90 (0.70, 1.17)	0.904
White cell count (per 109 cells/l)				
≤7.75	197.00	0.53 (0.43, 0.63)	Reference	
>7.75	150.00	0.38 (0.32, 0.44)	1.39 (1.09, 1.76)	0.008
Percentage of neutrophils				
≤0.6825	208.00	0.54 (0.44, 0.64)	Reference	
>0.6825	139.00	0.35 (0.29, 0.41)	2.39 (1.87, 3.05)	<0.001
Neutrophil count (per 109 cells/l)				
≤4.685	166.00	0.55 (0.43, 0.67)	Reference	
>4.685	181.00	0.38 (0.32, 0.44)	1.54 (1.21, 1.96)	<0.001
Percentage of lymphocytes				
≤0.2255	149.00	0.32 (0.27, 0.37)	Reference	
>0.2255	198.00	0.57 (0.46, 0.68)	0.45 (0.35, 0.57)	<0.001
Lymphocyte count (per 109 cells/l)				
≤2.005	230.00	0.42 (0.36, 0.48)	Reference	
>2.005	117.00	0.55 (0.42, 0.68)	0.63 (0.49, 0.83)	<0.001
Percentage of monocytes				
≤0.0765	173.00	0.58 (0.46, 0.70)	Reference	
>0.0765	174.00	0.35 (0.30, 0.40)	1.77 (1.39, 2.25)	<0.001
Monocyte count (per 109 cells/l)				
≤0.5964	157.00	0.68 (0.50, 0.86)	Reference	
>0.5964	190.00	0.29 (0.25, 0.34)	2.45 (1.91, 3.15)	<0.001
Red blood cell count (per 1012 cells/l)				
≤4.355	132.00	0.26 (0.22, 0.30)	Reference	
>4.355	215.00	0.59 (0.48, 0.70)	0.46 (0.36, 0.59)	<0.001
Hemoglobin (g/l)				
≤129.6	134.00	0.23 (0.20, 0.26)	Reference	
>129.6	213.00	0.61 (0.49, 0.73)	0.42 (0.33, 0.54)	<0.001
Platelet count (per 109 cells/l)				
≤287	261.00	0.50 (0.42, 0.58)	Reference	
>287	86.00	0.36 (0.28, 0.44)	1.58 (1.21, 2.07)	<0.001
Albumin (g/l)				
≤40.55	95.00	0.24 (0.20, 0.28)	Reference	
>40.55	252.00	0.55 (0.46, 0.64)	0.40 (0.30, 0.51)	<0.001
Globulin (g/l)				
≤31.95	168.00	0.56 (0.44, 0.68)	Reference	
>31.95	179.00	0.37 (0.31, 0.43)	1.65 (1.29, 2.10)	<0.001
Albumin/globulin ratio				
≤1.34	160.00	0.34 (0.29, 0.39)	Reference	
>1.34	187.00	0.57 (0.45, 0.69)	0.50 (0.39, 0.64)	<0.001
Total protein (g/l)				
≤65.5	28.00	0.25 (0.17, 0.33)	Reference	
>65.5	319.00	0.48 (0.42, 0.54)	0.49 (0.33, 0.73)	<0.001
AST (u/l)				
≤32.8	274.00	0.52 (0.44, 0.60)	Reference	
>32.8	73.00	0.25 (0.20, 0.30)	2.27 (1.71, 3.00)	<0.001
ALT (u/l)				
≤54.5	313.00	0.49 (0.42, 0.56)	Reference	
>54.5	34.00	0.24 (0.18, 0.30)	1.69 (1.15, 2.48)	0.008
AST/ALT				
≤1.21	216.00	0.52 (0.43, 0.61)	Reference	
>1.21	131.00	0.37 (0.30, 0.44)	1.44 (1.12, 1.83)	0.004
ALP (u/l)				
≤86.95	177.00	0.63 (0.48, 0.78)	Reference	
>86.95	170.00	0.29 (0.25, 0.33)	2.42 (1.89, 3.09)	<0.001
GGT (u/l)				
≤66	274.00	0.54 (0.45, 0.63)	Reference	
>66	73.00	0.19 (0.16, 0.22)	2.35 (1.77, 3.11)	<0.001
LDH (u/l)				
≤186.5	125.00	0.70 (0.46, 0.94)	Reference	
>186.5	222.00	0.33 (0.29, 0.37)	2.76 (2.10, 3.62)	<0.001
AFU (u/l)				
≤13.95	78.00	0.56 (0.48, 0.74)	Reference	
>13.95	269.00	0.43 (0.37, 0.49)	0.90 (0.68, 1.19)	0.447
TBIL (μmol/l)				
≤11.65	197.00	0.36 (0.31, 0.41)	Reference	
>11.65	150.00	0.60 (0.44, 0.74)	0.56 (0.43, 0.71)	<0.001
DBIL (μmol/l)				
≤2.885	117.00	0.38 (0.31, 0.45)	Reference	
>2.885	230.00	0.51 (0.43, 0.59)	0.70 (0.55, 0.90)	0.005
IBIL (μmol/l)				
≤7.935	195.00	0.34 (0.29, 0.39)	Reference	
>7.935	152.00	0.62 (0.47, 0.77)	0.56 (0.44, 0.71)	<0.001
HDL-C (mmol/l)				
≤0.995	119.00	0.28 (0.24, 0.32)	Reference	
>0.995	228.00	0.56 (0.46, 0.66)	0.49 (0.38, 0.63)	<0.001
LDL-C (mmol/l)				
≤3.2475	214.00	0.43 (0.37, 0.49)	Reference	
>3.2475	133.00	0.51 (0.39, 0.63)	0.72 (0.56, 0.93)	0.011
ApoA-I (g/l)				
≤1.1575	159.00	0.31 (0.26, 0.36)	Reference	
>1.1575	188.00	0.59 (0.47, 0.61)	0.47 (0.37, 0.60)	<0.001
Apo B (g/l)				
≤1.045	220.00	0.44 (0.38, 0.50)	Reference	
>1.045	127.00	0.51 (0.39, 0.63)	0.90 (0.70, 1.15)	0.401

Abbreviations: AST: aspartate aminotransferase; ALT: glutamic-pyruvic transaminase; ALP: alkaline phosphatase; GGT: glutamyltranspetidase; LDH: lactate dehydrogenase; AFU: alpha-fucosidase; TBIL: total bilirubin; DBIL: direct bilirubin; IBIL: indirect bilirubin; HDL-C: high-density lipoprotein cholesterol; LDL-C: low-density lipoprotein cholesterol; ApoA-I: Apolipoprotein A-I; Apo B: Apolipoprotein B;

* According to Union for International Cancer Control /American Joint Committee on Cancer (UICC/AJCC) TNM classification system (6th edition, 2002).

**Table 4:** Multivariate of clinicopathological characteristics

Variables	Adjusted HR (95%CI)	*P*
mNPC -SVM classifier		
Predicted low risk	Reference	
Predicted high risk	3.45 (2.59, 4.60)	< 0.001
Sex		
Male	Reference	
Female	0.90 (0.65, 1.24)	0.517
Age		
< 48 years	Reference	
≥ 48 years	1.06 (0.83, 1.36)	0.643
T Classification*		
T1-2	Reference	
T3-4	0.97 (0.85, 1.11)	0.642
N Classification*		
N0-1	Reference	
N2-3	1.13 (0.86, 1.48)	0.389
No. of metastatic organs		
Single	Reference	
Multiple	1.44 (0.82, 2.55)	0.206
No. of metastatic lesions		
Single	Reference	
Multiple	1.34 (0.85, 2.13)	0.210
Oligometastases		
No	Reference	
Yes	1.06 (0.71, 1.60)	0.765
Bony metastasis		
Absent	Reference	
Present	0.74 (0.48, 1.15)	0.185
Liver metastasis		
Absent	Reference	
Present	1.02 (0.66, 1.58)	0.936
Lung metastasis		
Absent	Reference	
Present	0.67 (0.41, 1.10)	0.116
Extraregional lymph node metastasis		
Absent	Reference	
Present	1.11 (0.88, 1.39)	0.380

* According to Union for International Cancer Control /American Joint Committee on Cancer (UICC/AJCC) TNM classification system (6th edition, 2002).

### The mNPC-SVM classifier is an outstanding indicator for the treatment choice

**Figure 3:**
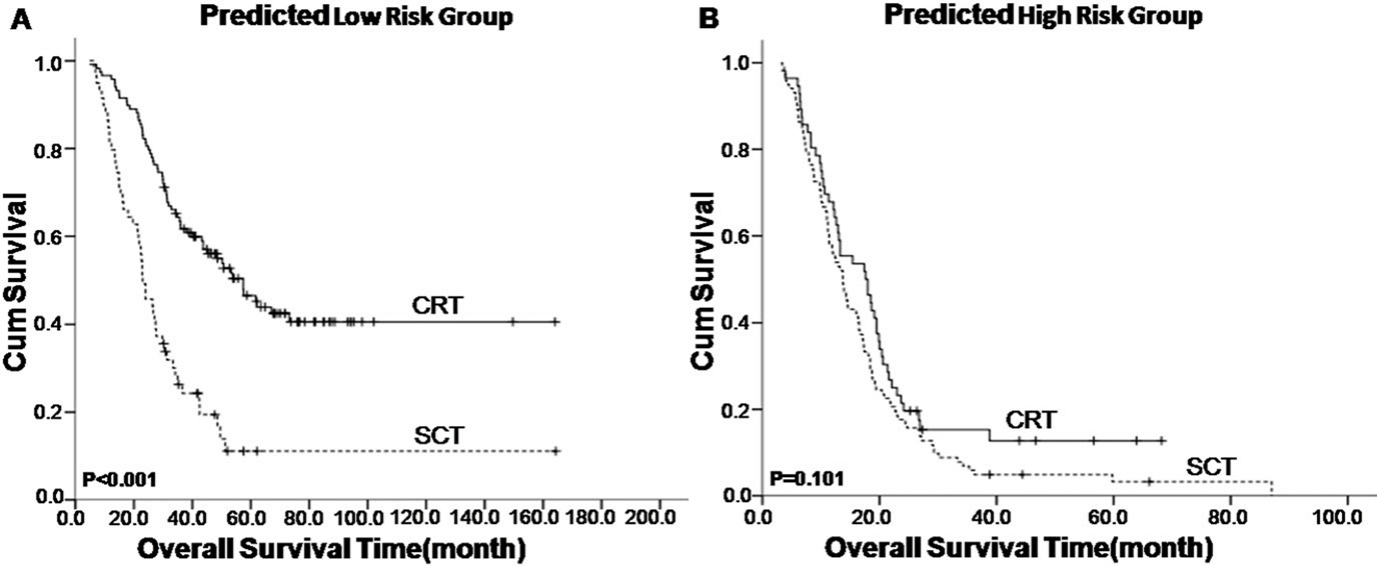
Kaplan–Meier survival analysis of treatment modality in nasopharyngeal carcinoma patients with synchronous metastases (mNPC) regarding the mNPC-SVM classifier CRT, chemoradiotherapy; SCT, systemic chemotherapy alone.

The efficacy of different treatment modalities was also investigated. Fifty-nine of the 182 patients identified as low risk were treated using systemic chemotherapy alone (SCT), 118 patients were treated using chemoradiotherapy (CRT), and 5 patients were treated using radiotherapy (RT). The 1-, 2-, 3- and 5-year OS rates in the SCT and CRT groups were 81.0% vs. 97.0%, 49.0% vs. 82.0%, 26.0% vs. 62.0%, and 10.0% vs. 47.0%, respectively (*P* < 0.001) (Figure 3A). A total of 102 of the 165 patients identified as high risk were treated using SCT, 56 patients were treated using CRT, and 7 patients were treated using RT. The 1-, 2-, 3- and 5-year OS rates in the SCT and CRT groups were 57.0% vs. 68.0%, 18.0% vs. 21.0%, 6.0% vs. 15.0%, and 3.0% vs. 12.0%, respectively (*P* = 0.101) (Figure 3B). In the validation cohort, CRT was also associated with significantly better survival outcomes than SCT in the M1a subgroup but not in the M1b subgroup (Supplemental Figure 3).

## DISCUSSION

TNM staging system is an excellent staging system for NPC patients. However, all patients with distant metastasis are staged as M1, which ignores the heterogeneity between patients. M1 staging may not be a good prognostic value for survival outcome or a good indicator of treatment choice for mNPC patients. Various specially designed prognostic models reportedly demonstrate enhanced prognostic value for patients with distant metastasis [8, 9]. However, no further aid for clinicians' choice of the most suitable treatment for these recurrent patients was evident. Therefore, we developed a novel ten-signature mNPC-SVM classifier to categorize patients with mNPC into high- and low-risk groups. The survival curves were distinctly separate between these two groups. The mNPC-SVM classifier was a significant independent prognostic factor for OS. ROC analyses also suggested that the mNPC-SVM exhibited a better prognostic value than the single indexes or markers. Chemotherapy plus radiotherapy was also associated with an enhanced survival benefit for M1 patients with low risk compared with systemic chemotherapy alone. However, a statistically significant difference was not observed in the CRT group compared with the SCT group for high-risk M1 patients with. M1 stage with low risk was defined as M1a, and M1 stage with high risk was considered M1b.

A single index or marker did not comprehensively reflect patient status. Therefore, our model is not a perfect prognostic model. Univariate analysis and traditional statistics are limited because they ignore the role of combinational potentials that may provide a good prediction of patient survival outcomes. Therefore, machine-learning methods were introduced in cancer classification and prediction because of their powerful capabilities in allowing inferences or decisions to be made that could not otherwise be made using conventional statistical methodologies [10-12]. Support vector machines (SVMs) are one of the most effective and widely used machine-learning techniques in the field of cancer prediction and prognosis [10, 13, 14]. The SVM algorithm creates a hyperplane that separates the data into two classes with the maximum margin, which is obtained by calculating the distance between the hyperplane and the closest examples or the margin. The application of different kernels to different data sets can dramatically improve the performance of an SVM classifier.

We designed the SVM models for mNPC via integration of three clinical indexes and seven hematological markers, which reflected the tumorigenesis phenotype of each patient macroscopically and microscopically. Therefore, a multi-biomarker-based model would provide more powerful efficacy for the prediction of patient outcome compared with single clinical index or hematological markers. The factors included in the SVM are used routinely in clinical practice, and they are easily available, which makes the model practical and convenient.

Palliative systemic chemotherapy is the major treatment modality for NPC patients with distant metastasis at diagnosis [15, 16]. However, the results of chemotherapy as an initial monotherapy in NPC patients with distant metastasis at diagnosis are not satisfactory. By contrast, radiotherapy or surgery for the primary lesions combined with systemic chemotherapy is beneficial for the survival outcome of these patients with distant metastases. Immunotherapy combined with radical nephrectomy resulted in better survival outcomes over time to progression (5 vs. 3 months, hazard ratio 0.60, 95% CI 0.36-0.97) and median duration of survival (11.1-17 vs. 7-8.1 months, *P* ≤ 0.05) [17, 18]. Morgan hypothesized that treatment directed against the primary tumor would retard the progression of existing metastases based on animal models and clinical observations [19]. Similarly, recent reports indicated that locoregional radiotherapy alone or combined with systemic chemotherapy was associated with improved survival of mNPC patients [7, 20]. However, these aggressive combination treatments may also result in adverse effects, especially treatment-related complications. Therefore, it is critical to identify patients who would benefit most from aggressive treatment modalities. The current study demonstrated that low-risk (M1a) patients exhibited better survival outcomes compared with high-risk (M1b) patients, but they were more suitable for systematic chemotherapy combined with locoregional radiotherapy. However, locoregional radiotherapy combined with chemotherapy failed to exert significant survival benefits in high-risk (M1b) patients compared to systemic chemotherapy alone.

This retrospective design had several potential limitations. First, although we included the majority of common clinical indexes and hematological markers in the current study, there were other relative indexes that were not included in the study. Therefore, the mNPC-SVM classifier may be further improved by the inclusion of some proven prognostic markers. Second, the efficacy of the combination treatment in high-risk mNPC patients cannot be guaranteed because of differences in the use of loco-regional radiotherapy and systemic chemotherapy in different institutions. Therefore, an optimal combination treatment schedule should be suggested for clinicians to provide the greatest survival benefit to mNPC patients.

In conclusion, the mNPC-SVM classifier exhibited better prognostic value the clinical indexes alone or hematological markers for mNPC patient survival, and this model may aid clinicians' selection of the most suitable treatment option for mNPC patients. However, we acknowledge that more studies are needed to validate this novel prognostic model.

## MATERIALS AND METHODS

### Patient data collection

The primary cohort of 347 mNPC patients for support vector machine (SVM) model development was derived from Sun Yat-sen University Cancer Center (SYSUCC) between January 2000 and December 2010. To examine the generalizability of the model, an validation cohort of 106 mNPC patients were included from SYSUCC between January 2011 and December 2012. Patients were excluded from the trial for any of the following criteria: 1) more than 3 months from the diagnosis of metastasis to pathological proof of NPC; 2) Karnofsky Performance Status score < 70; or 3) missing clinical/laboratory data. All patients were retrospectively classified into T1-4, N0-3, and M1 based on medical records using the Union for International Cancer Control /American Joint Committee on Cancer (UICC/AJCC) TNM classification system (6th edition, 2002). Bone metastasis was diagnosed based on patient history, physical examination, and imaging studies using a bone scan and/or positron-emission tomography/computed tomography and/or magnetic resonance imaging (MRI). The diagnosis of liver metastasis was based on histological evaluation, ultrasound or computed tomography of the abdomen. Lung metastasis was routinely determined using chest X-ray and/or computed tomography (CT). Pathological confirmation using a biopsy was performed when X-ray and/or CT was insufficient to confirm lung metastasis. The Clinical Ethics Review Board at Cancer Center of Sun Yat-sen University approved this study.

### Treatment

Cisplatin-based systemic chemotherapy was first provided to all patients as the basic treatment according to our institutional guideline for palliative treatment of mNPC. Definitive radiotherapy targeting of the primary tumor and its regional lymph nodes (locoregional radiotherapy, lrRT) was administered to some patients for local symptomatic treatment or as part of a multidisciplinary approach using two-dimensional conventional radiotherapy (2D-CRT) or intensity modulated radiotherapy (IMRT) as previously described [7, 21, 22].

### Patient follow-up

Clinical follow-up visits with each patient were scheduled on a semiannual basis. The follow-up division in the information department of SYSUCC ascertained patient's vital status, and follow-up information was updated until death from mNPC or the most recent follow-up, whichever occurred first. Causes of deaths were determined through death certificates, which were supplemented with medical records when necessary. The last follow-up dates were December 30, 2013 and June 30, 2015 for the primary and validation cohort respectively.

### Laboratory measurements

Blood biochemistries of all patients were determined using a Hitachi Automatic Analyzer 7600-020 (Hitachi High-Technologies, Tokyo, Japan). Complete blood count (CBC) was determined using a fully automated hematology analyzer Sysmex XE-5000 (Sysmex, Kobe, Japan). The inclusive criterion for the hematological indexes was the easily accessed variables in clinics.

### Selection of cut-off scores

The sensitivity and specificity of hematological markers as a predictor of death from mNPC within 2 years was plotted to generate a receiver operating characteristic (ROC) curve. We used 2-year OS as the outcome because median patient survival in this study was 22.6 months. ROC curves were used to select cut-off scores to dichotomize each predictor based on the score with the maximum area under the ROC curve (i.e., score nearest to a point on curve [0.0, 1.0] with maximum sensitivity and specificity).

### Statistical analysis

#### Univariate and multivariate analyses

Statistical analysis was performed using SPSS software (version 16.0, SPSS Inc., Chicago, IL). OS was defined as the time interval from the first diagnosis of metastatic NPC to death or the most recent follow-up. Univariate and multivariate analyses of variables were performed using Cox proportional hazards regression models. Actuarial OS was plotted against time using the Kaplan–Meier method, and differences between survival curves were compared using the log-rank test. The correlation of mNPC-SVM model with different clinicopathologic characteristics was evaluated by Spearman's rank correlation coefficient (r). The chi-square test was used to analyze differences in proportions. Statistical significance was defined as *P* < 0.05.

#### Prognostic analysis of survival status using machine learning modeling

CBC, blood biochemistry tests and clinical characteristics were combined as whole in this study to comprehensively characterize the survival status of mNPC. Classical machine learning techniques were employed to construct prognostic models and evaluate the prognostic capabilities of the combined factors (called *features* herein) on the models. A subset of features was obtained to achieve the best prognostic performance of patient survival patterns. Patients were dichotomized into two subgroups based on their median OS. Therefore, survival pattern analysis for mNPC was transformed to a binary classification problem, and the SVM model was used to construct prognostic classification models. The clinical parameters were further pruned to a compact yet informative subset to investigate informative variables that could distinguish low-risk patients from high-risk patients. This process is known as feature subset selection (FSS) [23-25], and we used the well-known Recursive Feature Elimination procedure based on SVM (RFE-SVM). The optimal subset with acceptable prognostic capabilities was obtained, and the SVM model was tested using a ten-fold cross-validation scheme. The entire patient set was dichotomized into two subgroups for risk assessment based on test results. The entire patient dataset was randomly divided into ten equal-sized groups in the ten-fold cross-validation model. Nine groups were used for model construction in each experiment, and the last group was used to test the model performance. The experiment was iterated until every group was tested, and the averaged performance was recorded to quantify the prognostic performance of the model.

## SUPPLEMENTARY MATERIAL FIGURES


