# Supplementary material for: Development of a ten-signature classifier using a support vector machine integrated approach to subdivide the M1 stage into M1a and M1b stages of nasopharyngeal carcinoma with synchronous metastases to better predict patients' survival
Source: Oncotarget. 2015 Nov 30;7(3):3645–57. doi: 10.18632/oncotarget.6436 (PMC4823134; doi:10.18632/oncotarget.6436)
Supplement: Supplementary file 1 [file oncotarget-07-3645-s001.pdf]

Development of a ten-signature classifier using a support vector machine integrated approach to subdivide the M1 stage into M1a and M1b stages of nasopharyngeal carcinoma with synchronous metastases to better predict patients' survival

Supplementary Material

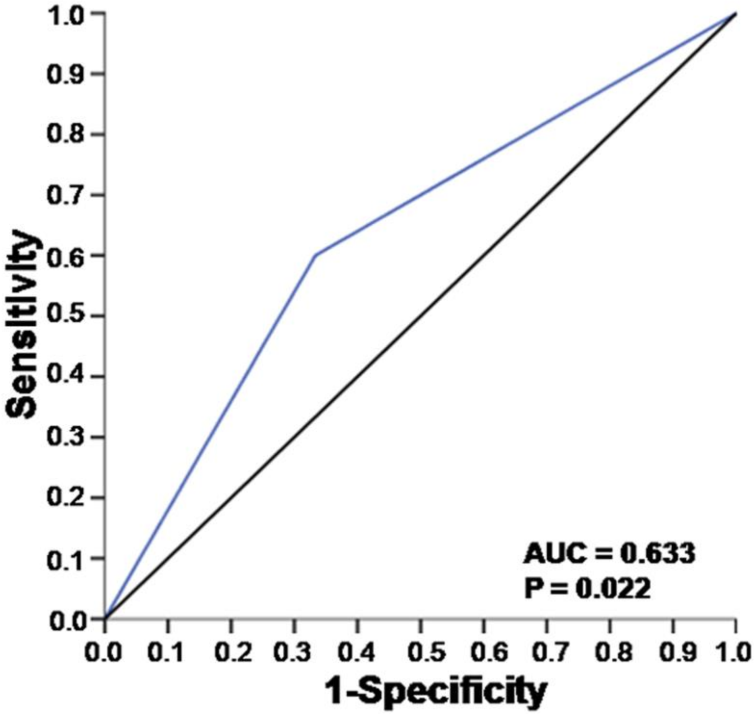

Supp. Figure 1. The receiver operating characteristic (ROC) curve for the mNPC-SVM classifier in the validation cohort.

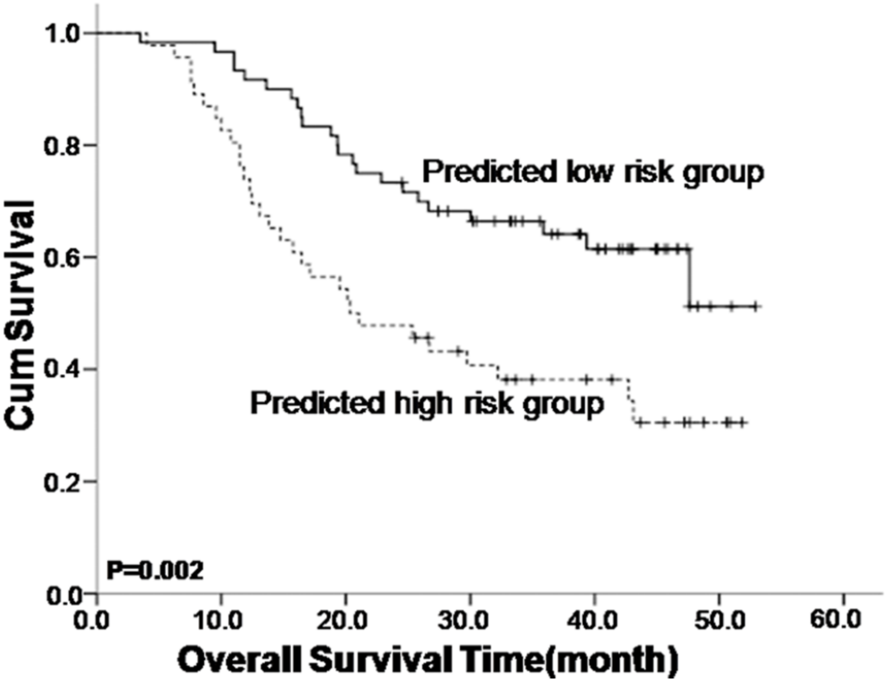

Supp. Figure 2. Kaplan–Meier survival analysis of the mNPC-SVM classifier in nasopharyngeal carcinoma patients with synchronous metastases (mNPC) in the validation cohort.

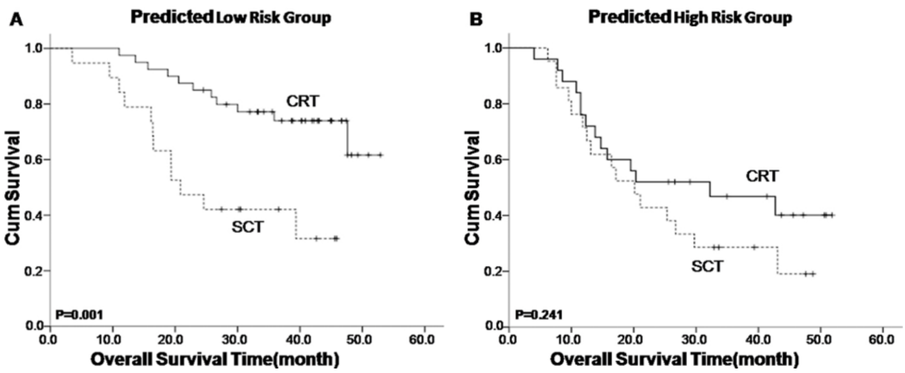

Supp. Figure 3. Kaplan–Meier survival analysis of treatment modality in nasopharyngeal carcinoma patients with synchronous metastases (mNPC) regarding the mNPC-SVM classifier in the validation cohort. CRT, chemoradiotherapy; SCT, systemic chemotherapy alone.
